# Prevalence of *Blastocystis* sp. in Morocco: Comparative assessment of three diagnostic methods and characterization of parasite forms in Jones’ culture medium[Fn FN1]

**DOI:** 10.1051/parasite/2023065

**Published:** 2023-12-20

**Authors:** Mounia Boutahar, Mourad Belaouni, Azeddine Ibrahimi, Rachid Eljaoudi, Tarik Aanniz, Mohammed Er-Rami

**Affiliations:** 1 Human Pathology, Biomedicine and Environment Laboratory, Faculty of Science and Technology of Fez B.P. 2202 – Route d’Imouzzer Fez Morocco; 2 Parasitology-Mycology Laboratory, Moulay Ismaïl Military Hospital Meknes 50007 Morocco; 3 Biotechnology Lab (MedBiotech), Bioinova Research Center, Rabat Medical & Pharmacy School, Mohammed V University in Rabat Rabat 10100 Morocco; 4 Mohammed VI Center for Research & Innovation (CM6) Rabat 10000 Morocco; 5 Mohammed VI University of Health Sciences Casablanca 20000 Morocco; 6 Emergency Department, Military Hospital Mohammed V Rabat 10000 Morocco; 7 Human Pathology, Biomedicine and Environment Laboratory, Faculty of Medicine and Pharmacy Fez 30070 Morocco

**Keywords:** *Blastocystis* sp., Jones’ medium, PCR, Sensitivity, Specificity, Morocco

## Abstract

Blastocystosis is an infection caused by *Blastocystis* sp., which colonizes the digestive tract of various hosts, including humans, although its pathogenicity is debated. It is crucial to detect and distinguish the different forms of *Blastocystis* to understand better its impact on human health and its epidemiological evolution. This study evaluated three diagnostic methods on 105 stool samples: direct examination, culture in Jones’ medium, and conventional PCR. PCR is considered the gold standard and revealed a high prevalence of *Blastocystis* (67.62%) compared to direct examination (20.95%) and culture in Jones’ medium (51.43%). Although the sensitivity of direct examination and culture was 31% and 76.1%, respectively, their specificity was 100%. No significant risk factors were identified. A statistically significant association was observed between *Blastocystis* infection and abdominal pain. Microscopic analysis revealed various morphological forms. Molecular diagnosis is an essential tool to determine the true prevalence of *Blastocystis*, and studying the different forms of this microorganism will contribute to a better understanding of its biological cycle and, therefore, the impact of this emerging infection on human health.

## Abstract (fr)

La blastocystose est une infection causée par *Blastocystis* sp., qui colonise le tractus digestif de divers hôtes, y compris l’homme, bien que son pouvoir pathogène soit débattu. Il est crucial de détecter et de distinguer les différentes formes de *Blastocystis* pour mieux comprendre son impact sur la santé humaine et son évolution épidémiologique. Cette étude a évalué trois méthodes de diagnostic sur 105 échantillons de selles : l’examen direct, la culture en milieu de Jones et la PCR conventionnelle. La PCR, considérée comme méthode de référence, a révélé une prévalence élevée de *Blastocystis* (67,62 %) par rapport à l’examen direct (20,95 %) et à la culture en milieu de Jones (51,43 %). Bien que la sensibilité de l’examen direct et de la culture soit respectivement de 31 % et 76,1 %, leur spécificité était de 100 %. Aucun facteur de risque significatif n’a été identifié. Une association statistiquement significative a été observée entre l’infection à *Blastocystis* et les douleurs abdominales. L’analyse microscopique a révélé diverses formes morphologiques. Le diagnostic moléculaire est un outil essentiel pour déterminer la véritable prévalence de *Blastocystis*, et l’étude des différentes formes de ce microorganisme contribuera à une meilleure compréhension de son cycle biologique et, par conséquent de l’impact de cette infection émergente sur la santé humaine.

## Introduction

Intestinal parasitic infections, including those caused by enteric protozoa, pose a significant global health challenge. Among these, *Blastocystis* sp. has gained attention as a prevalent microorganism often found in stool examinations. This anaerobic unicellular organism, present in various hosts, including humans with or without symptoms, is of interest [21, 43]. Some authors have reported that *Blastocystis* infection results in symptoms like diarrhea, abdominal pain, flatulence, nausea, and bloating [12, 24] and has links to conditions like irritable bowel syndrome, non-specific colitis, chronic inflammatory bowel disease, and urticaria [9]. Its diverse symptomatology challenges our understanding of intestinal parasitic infections. Detecting *Blastocystis* sp. relies on various methods: conventional microscopy, phase contrast, electron microscopy, culture, serodiagnosis, and advanced molecular techniques. Molecular tools have shown superior sensitivity and specificity [22, 35]. *Blastocystis* exhibits polymorphism, appearing as granular, vacuolar, cystic, and amoeboid forms, with the amoeboid form linked to symptoms [29, 40]. Although initial studies identified binary fission as its sole reproduction method [6], later studies revealed other modes such as budding, plasmotomy, and schizogony [16, 35, 70], adding complexity to its biology. *Blastocystis* sp. shows a global distribution, but the prevalence varies widely between countries, ranging from 7% to 50% in developed nations to 55% to 100% in developing countries [8, 26, 65]. Several studies suggest associations with risk factors like age, gender, personal hygiene, education, socioeconomic status, parasitic history, sanitation, and proximity to animals [12, 66]. The protozoan’s pathogenicity remains under debate, partly due to its genetic diversity, hinting at the existence of pathogenic and non-pathogenic subtypes. Currently, 40 subtypes, designated by numbers, have been identified in mammals and birds. Among these subtypes, 16 have been found in humans. Similar genetic diversity, not designated by numbers, has been identified in cold-blooded vertebrates. Subtypes and other genetic variants were all identified by comparing 18S rRNA gene sequences [18, 27, 48].

In Morocco, research on this enigmatic intestinal parasite is scarce. However, this study, conducted at the Parasitology-Mycology laboratory of the Moulay Ismail Military Hospital in Meknes, marks the country’s first comprehensive investigation. It aims to assess three diagnostic methods (direct examination, culture in Jones’ medium, and conventional PCR) for *Blastocystis* identification in stool samples from symptomatic and asymptomatic individuals. The study seeks to determine the most reliable diagnostic methods by comparing their sensitivity and specificity. Simultaneously, it explores risk factors and potential associations between *Blastocystis* infection and gastrointestinal symptoms. Additionally, the research delves into *Blastocystis* sp. various morphological forms, aiming to enhance understanding of its impact on human health.

## Materials and methods

### Ethics approval and consent to participate

This study obtained ethics approval from the Faculty of Medicine and Pharmacy of Fes for disease control and prevention, following established guidelines and regulations. Patients received oral explanations about the study’s objectives and procedures. Adult participants provided informed consent by signing the form, while participants under 20 years of age required parental authorization.

### Population studied

This analytical cross-sectional study was conducted at the Parasitology-Mycology laboratory of the Moulay Ismail Military Hospital in Meknes between November 2020 and September 2021. A standardized questionnaire including clinical, epidemiological, and socio-demographic data was completed for each participant. Subsequently, three random fecal samples were collected from each patient and then analyzed for the presence of intestinal parasites.

### Direct examination

All fecal samples in our study were divided into three parts. The first part was analyzed, immediately or at the latest within one hour after sample collection, by light microscopy of smears with physiological serum and Lugol’s iodine and after concentration by the MIF Bailanger technique to detect helminth eggs and larvae, cysts and trophozoites of protozoan parasites. The second part of each sample was stored at −20 °C until use for molecular analysis. The third part was cultured on Jones’ medium to identify the forms and mode of reproduction of *Blastocystis* sp.

### Culture in Jones’ medium

Approximately 50 mg of each fecal sample was inoculated into sterile screw-capped tubes containing 5 mL of Jones’ medium [20] supplemented with 10% horse serum. The tubes were placed under anaerobic conditions in BD GasPakTM EZ (BD) jars without using the pouches, which generate an anaerobic atmosphere, and incubated at 37 °C. These liquid cultures were subcultured every two days with a new complete Jones’ medium under ambient air conditions, then replaced in the anaerobic jars. The growth of *Blastocystis* was confirmed by microscopic observation of the culture for 14 days. Then, 200 μL of each positive sample was transferred to a 2 mL microtube and stored at a temperature of −20 °C for DNA extraction [2, 52].

### DNA extraction

Total genomic DNA from each *Blastocystis* isolate was extracted from stool samples using a DNA extraction kit (Nextractor NX-48S viral NA kit, Genolution, Seoul, South Korea), according to the manufacturer’s instructions. The quantity and quality of DNA were specified using a nano spectrophotometer (NanoVue Plus). The final DNA elution was created in 50 μL of elution buffer and stored at −20 °C until further use [22].

### PCR amplification

The amplification of *Blastocystis*-specific DNA was carried out using the following primers: forward (Blast 505–532: 5′–GGA GGT AGT GAC AAT AAA TC–3′) and reverse (Blast 998 – 1017: 5′–TGC TTT CGC ACT TGT TCA TC–3′). These primers generated approximately a 500 bp fragment [5].

The PCR conditions consisted of an initial activation step at 95 °C for 5 min, followed by 35 cycles at 95 °C for 30 s, 55 °C for 30 s, and 72 °C for 2 min, with a final extension step at 72 °C for 7 min.

PCR amplification reactions were conducted in a 96-well thermal cycler (MultiGene™ OptiMax Thermal). The PCR products were separated by electrophoresis on a 1.5% agarose gel containing SYBR™ Safe DNA Gel (Invitrogen™, Thermo Fisher Scientific, Waltham, MA, USA) and then visualized under UV transillumination. PCR-positive amplicons were stored at −20 °C for subsequent genotyping and sequencing.

### Statistical analysis

Data were input into Microsoft Excel and analyzed with IBM SPSS Statistics 26.0. A Pearson chi-square test (χ²) was used to explore associations between *Blastocystis* infection and clinical, epidemiological, and socioeconomic factors, with a significance level set at *p* ≤ 0.05.

Model performance in distinguishing positive from negative cases was evaluated for accuracy through sensitivity, specificity, and positive and negative predictive values (PPV and NPV). The overall discrimination ability was assessed by the area under the receiver operating characteristic (ROC) curve. Additionally, 95% confidence intervals (95% CI) were calculated for accuracy parameters and the ROC curve area.

## Results

The population of our study was composed of 105 participants, with 56 men and 49 women, randomly selected from a group of patients, including both symptomatic (*n* = 55) and asymptomatic (*n* = 50) individuals. The mean age of the participants was 37.07 ± 17.126 years, with an age range of 2–71 years.

In the present study, direct examination of smears with saline and Lugol’s iodine revealed a total prevalence rate of parasitic infections of 34.29% (36/105). The most common parasite was *Blastocystis* sp., present in 20.95% of patients (22/105), followed by *Dientamoeba fragilis*, *Entamoeba coli*, *Endolimax nana*, *Chilomastix mesnilii*, *Pseudolimax butschlii*, and *Giardia intestinalis* with 8.57% (9/105), 5.71% (6/105), 2.86% (3/105), 1.90% (2/105), 1.90% (2/105), and 0.95% (1/105), respectively.

**Figure 1:**
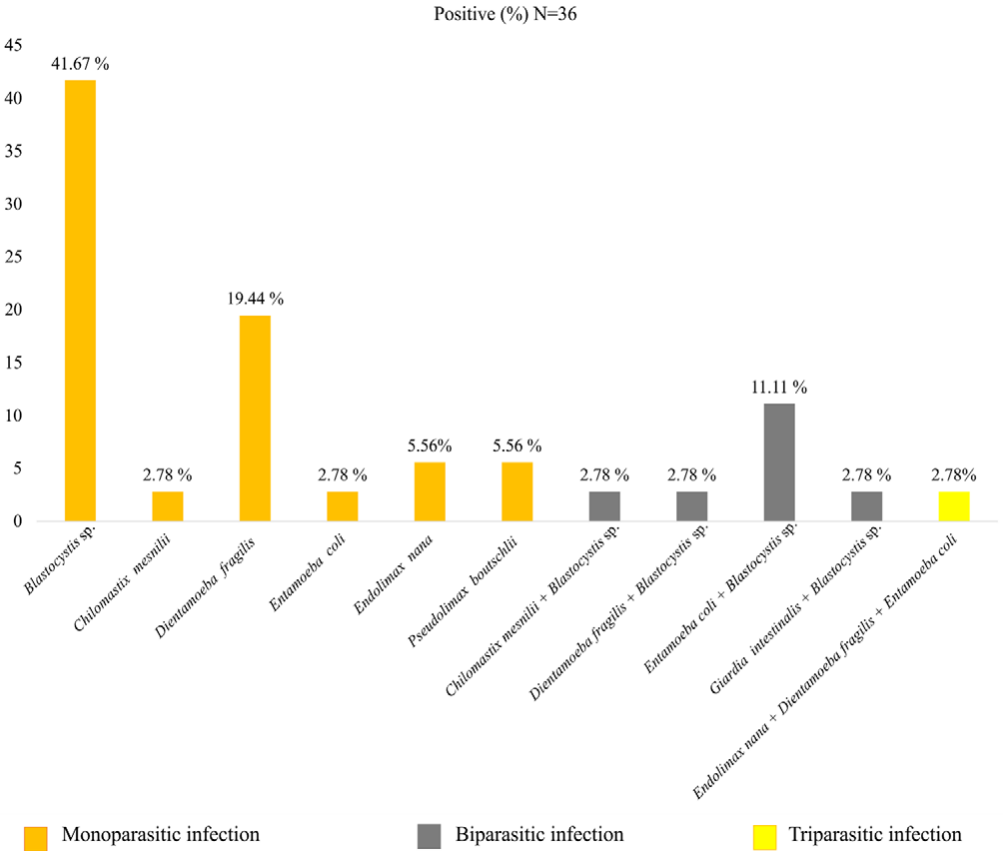
Dispersion of intestinal parasitic species among infected individuals.

**Figure 2:**
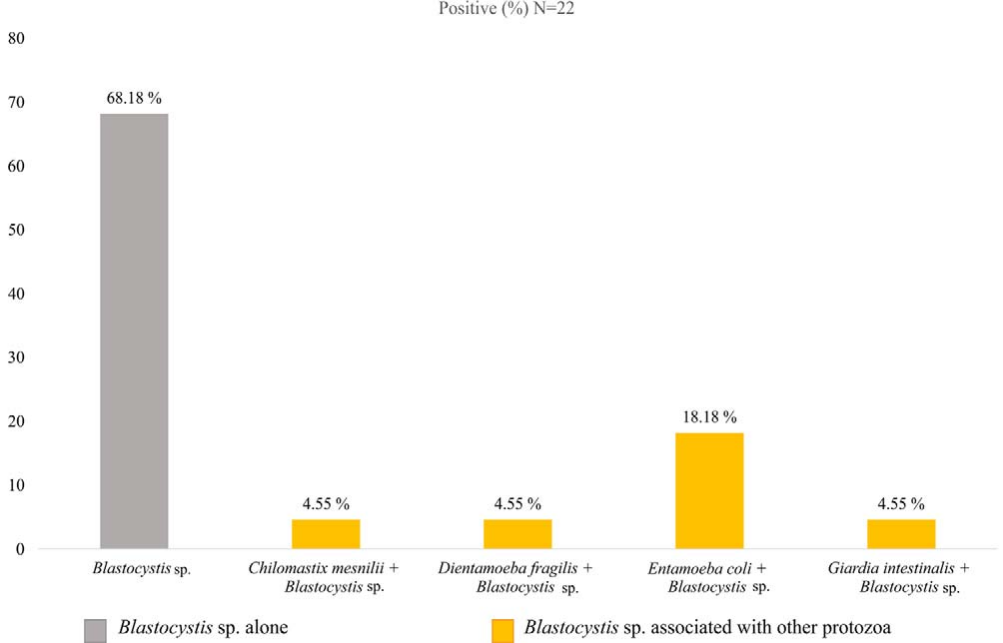
Occurrence of *Blastocystis* sp. infection on its own or in conjunction with other protozoan species.

These identified species are found alone (monoparasitism) in 77.78% of cases (28/36), in double association (biparasitism) in 19.44% (7/36), or triple association (triparasitism) in 2.78% (1/36). Mixed two-parasite infection between *Blastocystis* sp. and *Entamoeba coli* is the most common, accounting for 11.11% (4/36) (Fig. 1). Additionally, out of 36 stool samples, 15 (41.67%) were found to be infected with *Blastocystis* sp. only, while 7 (19.44%) had *Blastocystis* sp. in association with other parasites (Fig. 2).

**Figure 3:**
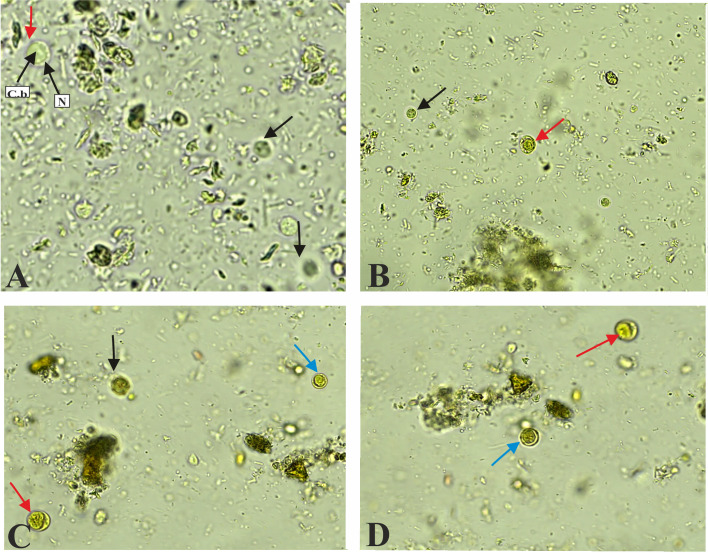
Various forms of *Blastocystis* sp. were observed under the light microscope during the direct examination of stool specimens. Panel A: Vacuolar form (red arrow) and cyst form (black arrow) of *Blastocystis* in an unstained wet mount. N: Nuclei situated at the periphery of the organism. C. b: Central body. Panels B, C, and D: Vacuolar form (red arrow), Granular form (blue arrow), and cyst form (black arrow) stained with Lugol’s iodine (×400).

**Figure 4:**
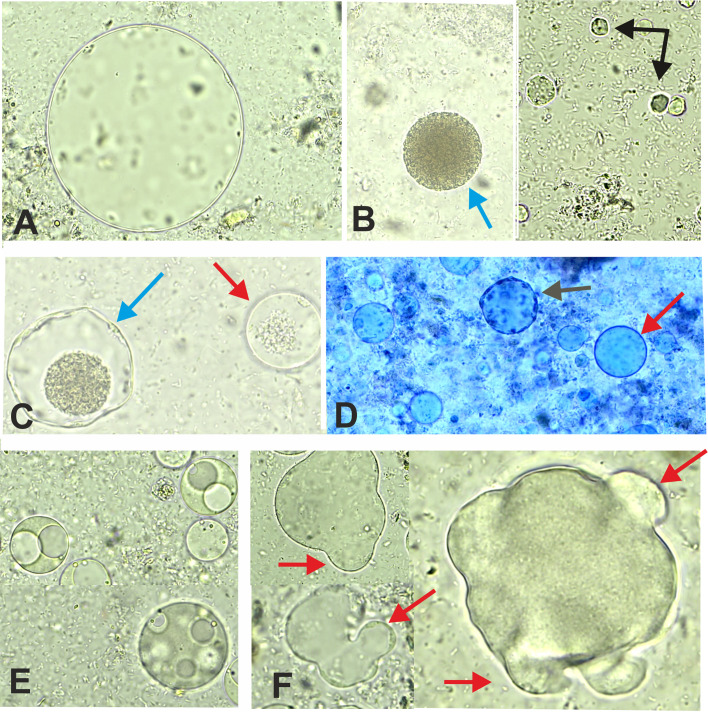
Observation under the light microscope (×400) of different forms of cultured *Blastocystis* sp. in Jones’ medium. Panel A: Different sizes of the vacuolar form. Panel B: Granular form (blue arrow) and cystic form (black arrow). Panel C: Granular form (blue arrow) and vacuolar form (red arrow). Panel D: Granular form (black arrow) and vacuolar form (red arrow) stained with methylene blue. Panel E: Illustrates the process of transformation of vacuolar cells into multivacuolar forms in culture, showing the division of the central vacuole into smaller vacuoles. Panel D: Different aspects of the amoeboid form with the presence of a single or several pseudopodia (red arrow).

**Figure 5:**
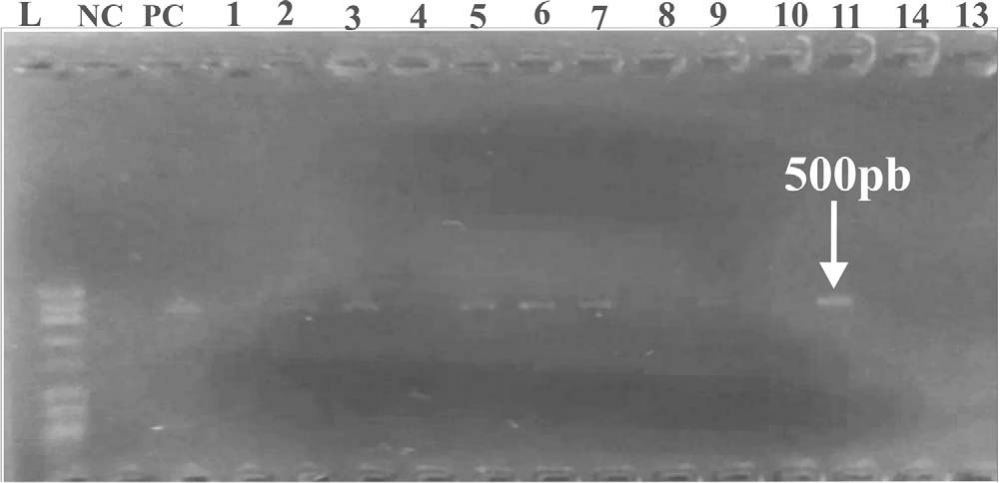
Representative gel image of PCR products from *Blastocystis* isolates. Lanes 1 to 13: *Blastocystis* isolates; lane NC: negative control; lane PC: positive control; DNA ladder – 50 bp.

In this study, the prevalence of *Blastocystis* sp. was determined by light microscopy, culture, and PCR. The results showed that PCR (Fig. 5) recorded the highest infection rate at 67.62% (71/105), followed by culture at 51.43% (54/105), while light microscopy showed the lowest rate at 20.95% (22/105).

PCR is considered to be the gold standard reference method for the detection of *Blastocystis* sp. due to its greater sensitivity and specificity. Direct examination showed a sensitivity of 31%, specificity of 100%, and accuracy of 53.33%, with “fair” agreement between tests. In contrast, Jones’ medium culture had a sensitivity of 76.1%, specificity of 100%, and accuracy of 83.81%, with a “substantial” level of agreement between assays (Table 4).

Table 1 presents statistical analysis results of sociodemographic factors such as age and gender in relation to the presence of *Blastocystis* sp. However, none of these factors revealed a statistically significant association (Table 1). Among the symptoms reported in our study, abdominal pain (*p* = 0.006) showed a strong statistically significant association with *Blastocystis* sp. infection (Table 3).

**Table 1:** Socio-demographic information of symptomatic and asymptomatic participants (*n* = 105) in the study.

Variables	Infection with *Blastocystis* sp.	Total (*n*%)	*p*-value	
	Positive (n%)	Negative (*n*%)		
Gender				0.956
Male	38 (53.5%)	18 (52.9%)	56 (53.3%)	
Female	33 (46.5%)	16 (47.1%)	49 (46.7%)	
Age groups (years)				0.329
2–11	4 (5.6%)	5 (14.7%)	9 (8.6%)	
12–21	5 (7%)	5 (14.7%)	10 (9.5%)	
22–31	12 (16.9%)	7 (20.6%)	19 (18.1%)	
32–41	24 (33.8%)	6 (17.6%)	30 (28.6%)	
42–51	11 (11.5%)	3 (8.8%)	14 (13.3%)	
52–61	8 (11.3%)	5 (14.7%)	13 (12.4%)	
62–71	7 (9.9%)	3 (8.8%)	10 (9.5%)	
Source of consumed water				0.683
Tap water	69 (97.2%)	33 (97.1%)	102 (97.1%)	
Mineral water	1 (1.4%)	0 (0%)	1 (1%)	
Well water	1 (1.4%)	1 (2.9%)	2 (1.9%)	
Restaurant or snack/week				0.196
0	46 (64.8%)	27 (79.4%)	73 (69.5%)	
1	16 (22.5%)	6 (17.6%)	22 (21%)	
2	9 (12.7%)	1 (2.9%)	10 (9.5%)	
Raw vegetables				0.455
Frequently	41 (57.7%)	17 (50%)	58 (55.2%)	
Rarely	30 (42.3%)	17 (50%)	47 (44.8%)	
Residency area				0.487
Rural	1 (1.4%)	0 (0%)	1 (1%)	
Urban	70 (98.6%)	34 (100%)	104 (99%)	

**Table 2:** Frequency of identified *Blastocystis* sp. using the PCR method among symptomatic and asymptomatic individuals.

		Symptoms	Total	*p*-value	
		Symptomatic	Asymptomatic		
PCR	Positive	40 (56.3%)	31 (43.7%)	71(100%)	0.241
	Negative	15 (44.1%)	19 (55.9%)	34 (100%)	
Total		55 (52.4%)	50 (47.6%)	105 (100%)	

**Table 3:** Association between positive cases of *Blastocystis* sp. and clinical symptoms.

Clinical symptoms	Infection with *Blastocystis* sp.		Total (*n*%)	*p*-value
	Positive (*n*%)	Negative (*n*%)		
Abdominal pain				0.006*
Yes	32 (45.1%)	6 (17.6%)	38 (36.2%)	
No	39 (54.9%)	28 (82.4%)	67 (63.8%)	
Constipation				0.824
Yes	5 (7%)	2 (5.9%)	7 (6.7%)	
No	66 (93%)	32 (94.1%)	98 (93.3%)	
Diarrhea				0.034
Yes	16 (22.5%)	2 (5.9%)	18 (17.1%)	
No	55 (77.5%)	32 (94.1%)	87 (83.9%)	
Flatulence				0.041
Yes	24 (33.8%)	5 (14.7%)	29 (27.6%)	
No	47 (66.2%)	29 (85.3%)	76 (72.4%)	
Anal itching				0.198
Yes	1 (1.4%)	2 (5.9%)	3 (2.9%)	
No	70 (98.6%)	32 (94.1%)	102 (97.1%)	
Skin rash				0.323
Yes	2 (0%)	0 (0%)	2 (1.9%)	
No	69 (100%)	34 (100%)	103 (98.1%)	
Fatigue				0.063
Yes	1 (1.4%)	3 (8.8%)	4 (3.8%)	
No	70 (98.6%)	31 (91.2%)	101 (96.2%)	
Weight loss				0.591
Yes	1 (1.4%)	1 (2.9%)	2 (1.9%)	
No	70 (98.6%)	33 (97.1%)	103 (98.1%)	

* Statistically significant *p* value (< 0.05).

**Table 4:** Sensitivity and specificity of different diagnostic methods for *Blastocystis* sp. using the PCR method as the gold standard.

	Light microscope	Jones’ medium culture
Sensitivity	31%	76.1%
Specificity	100%	100%
PPV	100%	100%
NPV	40.96%	66.67%
Accuracy	53.33%	83.81%
Kappa agreement	0.225	0.673

Microscopic analysis in the fresh state and after cultivation in Jones’ medium revealed the presence of different sizes and shapes of *Blastocystis*, as well as its different modes of reproduction. The vacuolar form was the most frequently observed morphological form of the parasite. However, our *in vitro* culture isolates also exhibited vacuolar, granular, amoeboid, cystic, and multivacuolar forms (Figs. 3 and 4).

The vacuolar form (Fig. 3) was the most common morphological form of the parasite in fresh smears, as well as in culture (Fig. 4). It appeared as a round or oval shape, containing a central body occupying 70% to 90% of the cytoplasm by pushing the nuclei toward the cytoplasmic membrane. The granular form (Figs. 3 and 4) was also the most frequently observed morphotype and was similar in size and shape to the vacuolar form, except for the presence of granules in the central body.

The multivacuolar form (Fig. 4) observed in our study appeared as an irregular round cell, relatively small in size compared to the vacuolar and granular forms, and was characterized by the presence of several small vacuoles separating the cytoplasm. Figure 4 illustrates the process of transformation of vacuolar cells into multivacuolar forms in culture, showing the division of the central vacuole into small vacuoles.

The amoeboid form highlighted in our study was detected only by the culture technique, this form appeared as an irregular oval cell with one or more pseudopodia, as well as a large central body (Fig. 4). This study also revealed the presence of the cystic form in fresh stools as well as in older cultures (Figs. 3 and 4). This form exhibits high refractive power in optical microscopy, its size appears smaller than other forms, and generally takes a rounded or oval shape, containing 1–4 nuclei.

## Discussion

In this study, parasitological stool examination (PSE) revealed an overall infection rate of 34.29%, similar to rates in Algeria (33.3%) [43], Colombia (36.6%) [34], Brazil (38.2%) [54], and Morocco (44%) [7]. In contrast, lower rates were found in Italy (13.24%) [38] and France (17%) [30], while much higher rates were reported in Egypt (72%) [17], Lebanon (85%) [33] and Nigeria (97%) [19].

*Blastocystis* sp. was the most common parasite with 20.95%, followed by *Dientamoeba fragilis* with 8.57%, and *Entamoeba coli* with 5.71%. These results are consistent with global data, with *Blastocystis* often being the most common parasite in both symptomatic and asymptomatic individuals [21, 43]. *Dientamoeba fragilis* and *Entamoeba coli* are often the second most widespread parasites [7, 30].

*Blastocystis* is found alone or in co-infections. Double associations were the most common, with *Blastocystis* sp. and *Entamoeba coli* being the most prevalent co-infection [34, 38]. Such co-infections are explained by shared modes of transmission. Notably, although *Entamoeba coli* is non-pathogenic [45], its presence indicates insufficient hygiene, posing a potential risk of transporting other similarly transmitted pathogenic parasites, bacteria, or viruses [21, 67].

In this study, 105 human stool samples were analyzed by microscopy, culture, and PCR for the detection of *Blastocystis*. PCR exhibited the highest positivity rate at 67.62%, followed by Jones’ medium culture at 51.43%, while optical microscopy had the lowest detection rate at 20.95%. Microscopy showed 31% sensitivity, 100% specificity, and “fair” agreement with other tests. In contrast, culture displayed 76.1% sensitivity, 100% specificity, and “substantial” agreement. Jones medium might provide an alternative for the detection of *Blastocystis* in resource-poor countries, where expensive PCR setups are not available.

Our findings align with previous studies that have shown that molecular methods are more reliable than culture and microscopy for *Blastocystis* detection [22, 35]. However, some rare studies suggest the utility of culture over PCR [41, 61]. Despite PCR’s advantages, its cost hinders routine clinical detection, especially in developing countries.

*Blastocystis* prevalence in humans is a matter of public health interest, but rates must be interpreted cautiously due to diagnostic method variations. Our PCR-based study reports a 67.62% prevalence. Similar diagnostic methods in Senegal, Syria (100%), Nigeria (84%), and Qatar (71.1%) recorded high positivity rates [3, 8, 12, 39]. Meanwhile, moderate rates were observed in Lebanon (63%), Spain (35.2%), and Italy (31.08%) [15, 33, 37]. France (18.1%) and Saudi Arabia (10.5%) reported relatively lower rates [12, 32].

In neighboring countries, optical microscopy revealed *Blastocystis* rates of 32.1% in Algeria, 22.1% in Libya, and 13% in Tunisia [1, 2, 44]. The primary mode of *Blastocystis* transmission is the fecal-oral route, often occurring through contact with contaminated objects, foods, and drinking water, and even with contact with infected animals [12, 31, 66].

Several risk factors, including age, gender, personal hygiene, education level, socio-economic status, parasitosis history, and health infrastructure access, have been associated with *Blastocystis* infection [11, 66]. However, our study showed no significant association between *Blastocystis* sp. and the risk factors considered in this research (Table 1).

The detection of *Blastocystis* in individuals, both symptomatic and asymptomatic, from various infection sources (zoonotic, environmental, and human), along with its genetic diversity, suggests the presence of pathogenic and non-pathogenic isolates [10, 14]. Common gastrointestinal symptoms associated with this infection include diarrhea, abdominal pain, and non-specific symptoms like nausea, vomiting, fatigue, and flatulence [12, 24].

Researchers like Matovelle *et al.* (2022) and A. Mohamed *et al.* (2017) have linked *Blastocystis* to gastrointestinal symptoms, particularly in immunocompromized individuals like AIDS and cancer patients, where it acts as an opportunistic pathogen [28, 31]. A recent study by AL-berfkani (2022) showed a significant association between *Blastocystis* infection and irritable bowel syndrome [4].

Our study revealed a strong statistically significant association between *Blastocystis* sp. infection and abdominal pain (Table 3). These results corroborate those of other studies [17, 28].

Despite numerous clinical and epidemiological studies, the pathogenicity of *Blastocystis* sp. remains a subject of debate, further complicated by the discovery of new subtypes. Currently, 40 subtypes (STs): ST1 to ST17, ST21, and ST23 to ST44 have been identified [18, 27, 48], with 16 detected in humans, including ST1 to ST10, ST12, ST14, ST16, ST23, ST35, and ST41 [18, 23, 27, 34]. Other subtypes, not designated by numbers were found in cold-blooded vertebrates. This diversity of subtypes raises questions about their potential roles and impacts on human health. To better understand their pathogenic potential, it is recommended to conduct specific genetic analyses at these levels [63]. Given that it is an indicator of poor orofecal hygiene, it is difficult to confirm whether these symptoms are due to the protozoan itself or to an enteropathogenic bacterium with the same mode of transmission. Many publications report no association between clinical symptoms and carriage of *Blastocystis* sp. Some authors have even suggested that this protozoan is an indicator of a healthy gut [11, 42].

Note that *Blastocystis* sp. exhibits a wide range of shapes and various morphologies documented in the literature, including vacuolar, granular, avacuolar, multivacuolar, amoeboid, and cystic forms [29, 47, 69]. Tan (2004) and Suresh *et al.* (2009) also identified a distinct pre-cystic form [53, 57]. Our study recognized some of these forms, including vacuolar, multivacuolar, granular, amoeboid, and cystic.

The vacuolar form (Fig. 3) was the most common morphological form of the parasite in direct smears, as well as in culture (Fig. 4) [29, 68]. In 2018, Thergarajan *et al.* demonstrated that the vacuolar form transforms into the granular form [62]. The granular form contains three types of granules: metabolic, reproductive, and lipid [55, 71].

Stenzel *et al.* (1991) found multivacuolar forms in human stools, with thicker surface layers in fresh samples than those cultured in the laboratory [49, 50]. These forms may serve as developmental stages of the parasite within the host [25] and as intermediate stages in the differentiation of fecal cysts into vacuolar forms [49]. Conversely, Boreham and Stenzel (1993) proposed that *in vivo* intestinal vacuolar forms transform into multivacuolar forms in stools [6], a theory corroborated by Singh *et al.* (1995) [47].

The amoeboid form is rarely reported and is mainly seen in symptomatic patients and in older cultures, but rarely in fresh stool samples [29, 69]. Studies on its morphology contain conflicting information [58, 72], probably due to genetic variations in *Blastocystis* isolates [56, 59]. While the amoeboid form has pseudopodia, no observable movements are reported, and their function remains unclear [46]. It might serve as an intermediate stage between vacuolar and cystic forms [47, 58] and potentially play a role in pathogenesis [40, 59].

Some cysts have thick outer layers [13], and deep pore-like openings [51, 53]. The cystic form is the parasite’s transmissible and resilient form, surviving harsh conditions like water, air, and temperature variations [36, 58, 69]. Singh *et al.*, 1995 suggested two cyst types: thick-walled cysts for external transmission *via* the fecal-oral route and thin-walled, auto-infectious cysts for multiplication in the intestinal tract [47, 50, 58, 68]. Several authors have reported that *Blastocystis* sp. can change its form over a short period because of external conditions, such as exposure to oxygen [64]. Subculturing of the protozoa in ambient air was a critical step that could influence the morphological forms observed in our study.

With regard to the reproductive process of *Blastocystis* sp., binary fission is the most commonly observed method of reproduction [50, 58]. In addition to this method, some studies have suggested other modes such as budding, plasmotomy, and schizogony-type reproduction. These results are consistent with several previous studies [16, 35, 70]. This diversity of reproductive modes has caused controversy, as binary division alone cannot fully account for the observed high growth rate in this parasite [60]. Additionally, the lack of electron microscopy results and the use of stress-induced conditions, such as heat stress [62] and metronidazole treatment, also contributed to these controversies [16].

However, a study conducted by Yamada and Yoshikawa in 2012, which employed both light and electron microscopy techniques in addition to DNA staining, provided evidence that binary fission, budding, and plasmotomy are the primary modes of reproduction in *Blastocystis* sp. [68].

## Conclusion and perspectives

In summary, our study highlights the effectiveness of PCR in detecting *Blastocystis* sp., surpassing direct examination and Jones’ medium culture. While we did not find significant associations between *Blastocystis* infection and the tested risk factors, further research in this area is crucial. Moreover, our study’s noteworthy findings indicate a significant connection between *Blastocystis* sp. infection and abdominal pain. Detailed microscopic examination of Jones’ medium cultures also unveiled diverse *Blastocystis* morphological forms. The establishment of a laboratory animal model could enable in-depth exploration of *Blastocystis* biological cycle, reproduction modes, subtype relationships, and potential pathogenicity. Additionally, comprehensive genetic studies may shed light on the complex interplay between symptom onset and *Blastocystis* sp. genetic diversity. Ultimately, future research will provide a comprehensive understanding of this intricate protozoal infection, paving the way for improved methods of detection, prevention, and treatment to enhance human health.
